# Surgical Realignment of a Dorsiflexed Metatarsal Fracture

**DOI:** 10.7759/cureus.9281

**Published:** 2020-07-19

**Authors:** Steven R Edwards, Simon E Smith

**Affiliations:** 1 Surgery, Australasian College of Podiatric Surgeons, Melbourne, AUS; 2 Podiatry, La Trobe University, Bundoora, AUS; 3 Podiatric Surgery, Australasian College of Podiatric Surgeons, Melbourne, AUS

**Keywords:** fracture, metatarsal, open reduction internal fixation, foot surgery techniques, foot injuries, fore foot

## Abstract

Metatarsal fractures are common injuries that routinely present to outpatient clinics. Whilst usually amenable to conservative care, there is controversy regarding treatment when the fracture results in significant misalignment. In this case report, a 54-year-old female recreational basketball player who sustained a second metatarsal fracture that had healed in a dorsiflexed position in relation to the adjacent metatarsals was referred for a surgical opinion. She had experienced worsening overload pain to her third metatarsophalangeal joint (MTPJ). Open reduction with internal fixation (ORIF) via a 6-hole locking plate was employed to reduce the fracture misalignment and re-establish the metatarsal parabola. She enjoyed an uneventful recovery with a full return to her sporting activities. ORIF with locking plate may be an acceptable technique for reducing displaced metatarsal fractures and re-establishing the metatarsal parabola.

## Introduction

Metatarsal fractures are common injuries that frequently present to outpatient clinics [1,2]. Although usually amenable to conservative care, surgery may be required when there is significant misalignment. When to perform surgery and the type of surgical intervention for these injuries is controversial [3].

The success of non-operative treatment appears to decrease as the degree of misalignment increases, yet there is no clear guideline regarding the degree of misalignment that requires surgical intervention. Current research recommends surgical repair when the fracture is displaced by 3 mm, or angled by 10 degrees [3-6]. Most authors agree that if alignment cannot be achieved conservatively, then open reduction with internal fixation (ORIF) is indicated - especially if the fracture segments are misaligned in the sagittal plane or there is sequential metatarsalgia, overloading of the adjacent metatarsals, plantar callosities or digital deformities [2-3,7-9].

We present a case report of a second metatarsal fracture that had healed in a dorsiflexed position 7 mm higher than the adjacent metatarsal. This resulted in worsening pain and overload symptoms to the third metatarsal head.

## Case presentation

A 54-year-old female high school mathematics teacher was referred to our surgical clinic with a dorsiflexed and shortened right second metatarsal after it was fractured during a social basketball game where a teammate landed directly onto the top of her foot. The fracture had healed following prolonged conservative care yet the misaligned position of the metatarsal resulted in worsening transfer overload pain to her third metatarsophalangeal joint (MTPJ). We measured the elevation of her misaligned second metatarsal head to be 7 mm superior to the third.

Her medical history was uneventful with only a past episode of transient post-natal depression two decades prior. She did not take any current medications and reported a slight erythematous reaction to Tegaderm® surgical dressing preoperatively during a finger ORIF in 2016. She was a non-smoker and had not experienced prior reactions to general anaesthetics.

Physical examination identified bony proliferation and prominence to the second metatarsal shaft at the apparent fracture site. There was a palpable depression underlying the plantar forefoot where the second metatarsal head should reside. The third MTPJ was prominent with recurrent and painful plantar callosity formation corresponding with her primary location of pain during weight-bearing. Her second digit had adopted a slightly elevated position.

**Figure 1:**
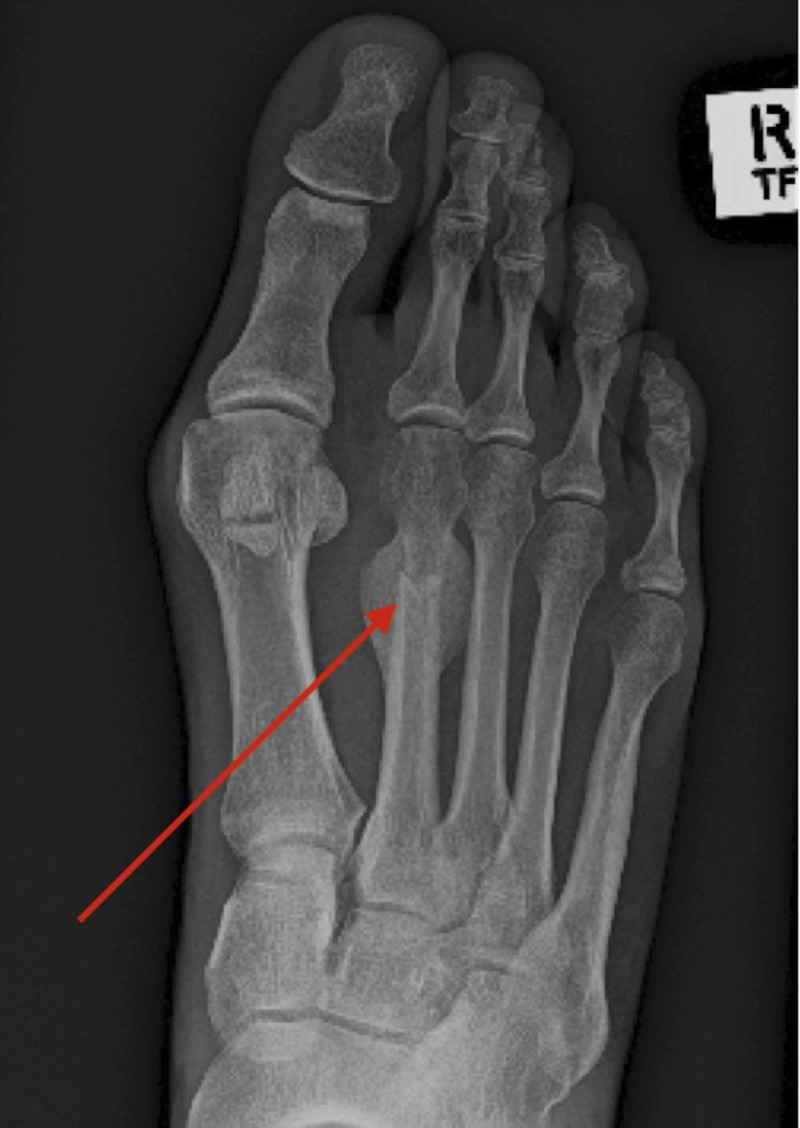
Anterior-Posterior radiograph showing the metatarsal fracture with osteophytic bone formation

**Figure 2:**
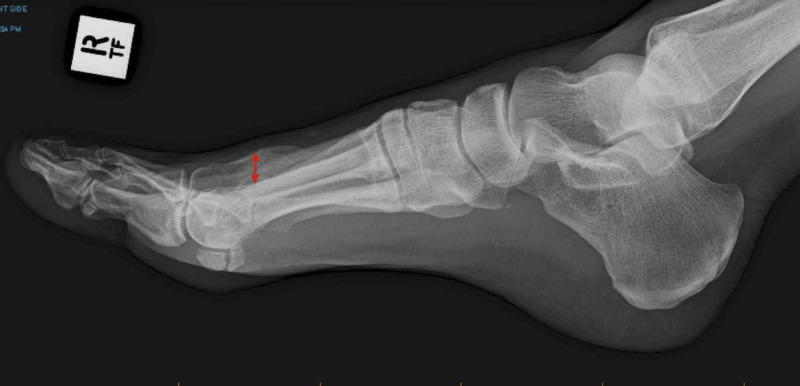
Lateral radiograph illustrating the elevation of the distal segment of the second metatarsal

Anterior-posterior and lateral radiographs exhibited a healed mid-shaft fracture of the right second metatarsal resulting in dorsal elevation and slight shortening, with some florid secondary bone healing surrounding the fracture site (Figures 1, 2). 

The patient was positioned supine on the operating table. A pneumatic calf tourniquet was applied around the muscle bulk of the right calf. A 20 mL 0.75% ropivacaine hydrochloride mixed with 4 mg dexamethasone sodium phosphate anterior ankle and second metatarsal field block was performed. The right lower extremity was prepped with a 10% povidone iodine solution and draped with sterile field and shut-out drapes. The right lower extremity was exsanguinated with a sterile esmarch bandage and the tourniquet was inflated to 250 mmHg. A dorsal longitudinal incision was performed, extending from the central midfoot to the proximal margin of the second MTPJ.

**Figure 3:**
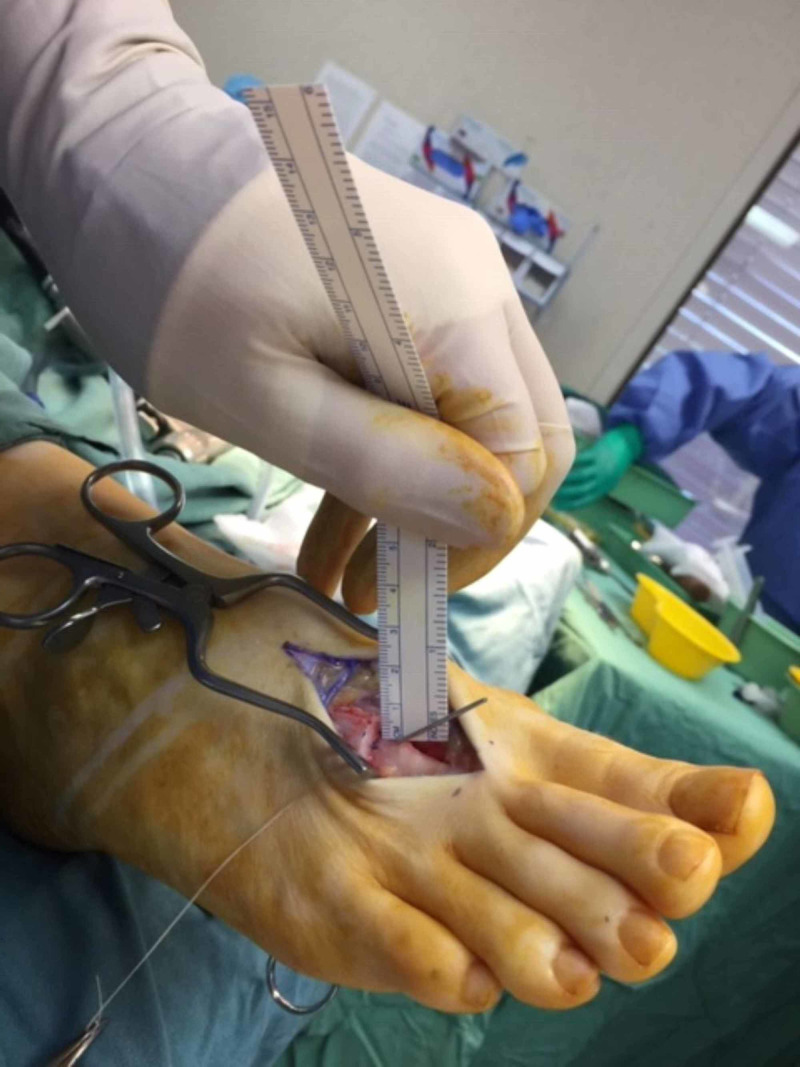
Reduction of the fracture to normal alignment with temporary Kirschner wire fixation

The incision was deepened through the superficial tissue using Metzenbaum scissors, an unloaded scalpel handle and saline-soaked gauze. Dissection was performed with care and maintenance to adequate haemostasis; vessels crossing the line of the incision were cauterised or retracted appropriately. The second metatarsal was identified and a longitudinal periosteal incision was performed. The lateral and medial periosteum of the second metatarsal was separated using a Freer elevator. A sagittal saw was employed to perform an oblique osteotomy of the mid-shaft of the second metatarsal, approximately 30 mm proximal to the second MTPJ. The orientation of the osteotomy was from superior-proximal to inferior-distal at an angle of approximately 40 degrees. The distal fragment was then manipulated (“slid”) distal-inferior by 4-5 mm and was temporarily fixated with a 1.2 mm Kirschner wire (Figure 3).

**Figure 4:**
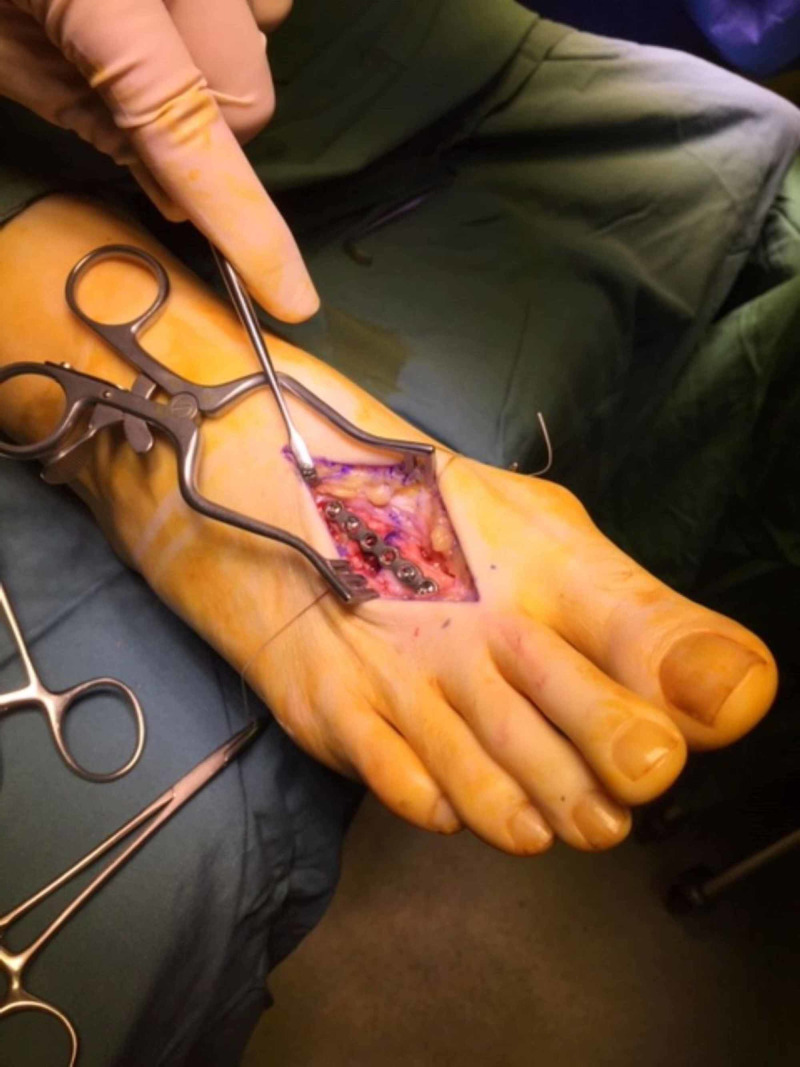
ORIF of the fracture site with a 6-hole 2.0 Osteomed® HPS locking plate ORIF, open reduction with internal fixation; HPS, Hand Plating System

Rongeurs were employed to resect the hyperostosis of the residuum of the proximal fragment and a 6-hole 2.0 Osteomed® Hand Plating System (HPS) locking plate was applied to the dorsal aspect of the second metatarsal (Figure 4). Her elevated second digit was reduced with a long-arm extensor tendon lengthening technique. Deep closure was achieved with 3-0 Vicryl running over-and-over suture, superficial fascia closure with 3-0 Vicryl over-and-over suture and skin closure with 3-0 Monocryl run-in subcuticular sutures. The wound was then dressed with the appropriate dressings and a fiberglass back-slab was applied with the foot maintained at 90-degrees to the lower leg. There was a good capillary refill of the digits of the right foot upon tourniquet deflation and completion of the procedure.

In order to maximise the patient's overall result, multi-modal treatment methods were implemented. These included careful surgical technique and preparation of clean wound edges, even approximation of the wound with minimal tension, careful reduction of the fracture and meticulous insertion and positioning of the fixation. Perioperative antibiotics and antiseptic dressings were implemented to reduce the risk of infection. Early compression therapy was also employed [10].

**Figure 5:**
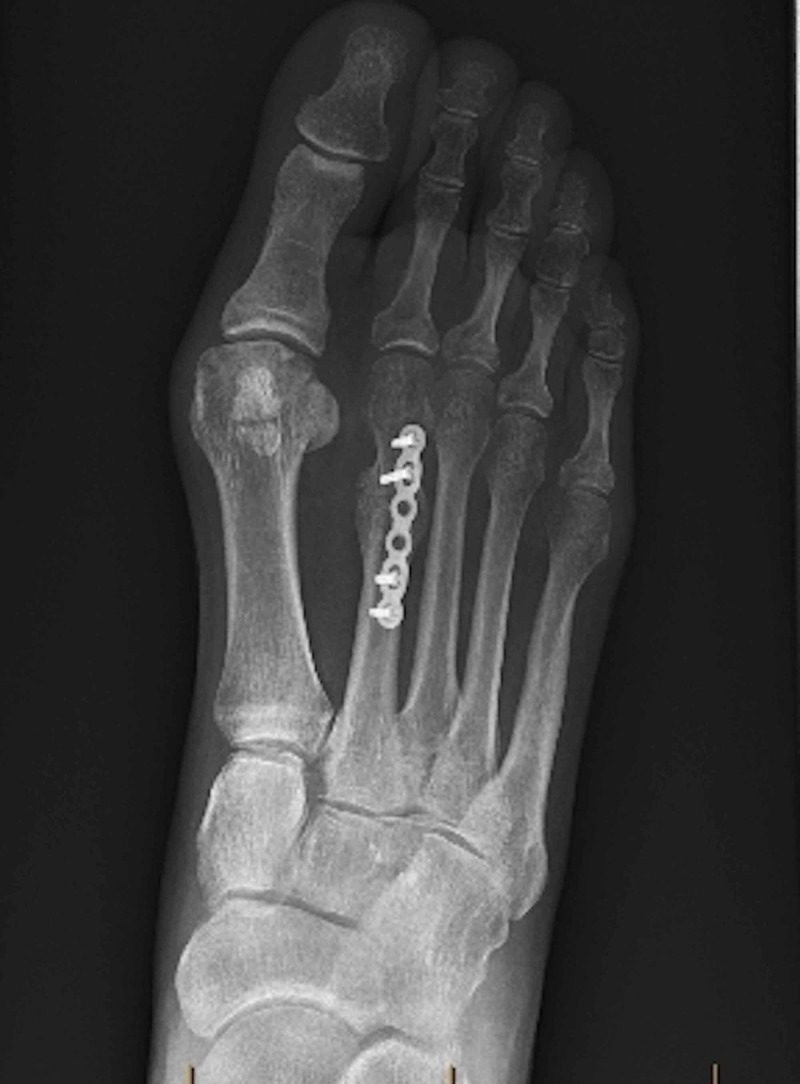
Post-operative anterior-posterior radiograph showing ORIF with re-establishment of the metatarsal parabola ORIF, open reduction with internal fixation

**Figure 6:**
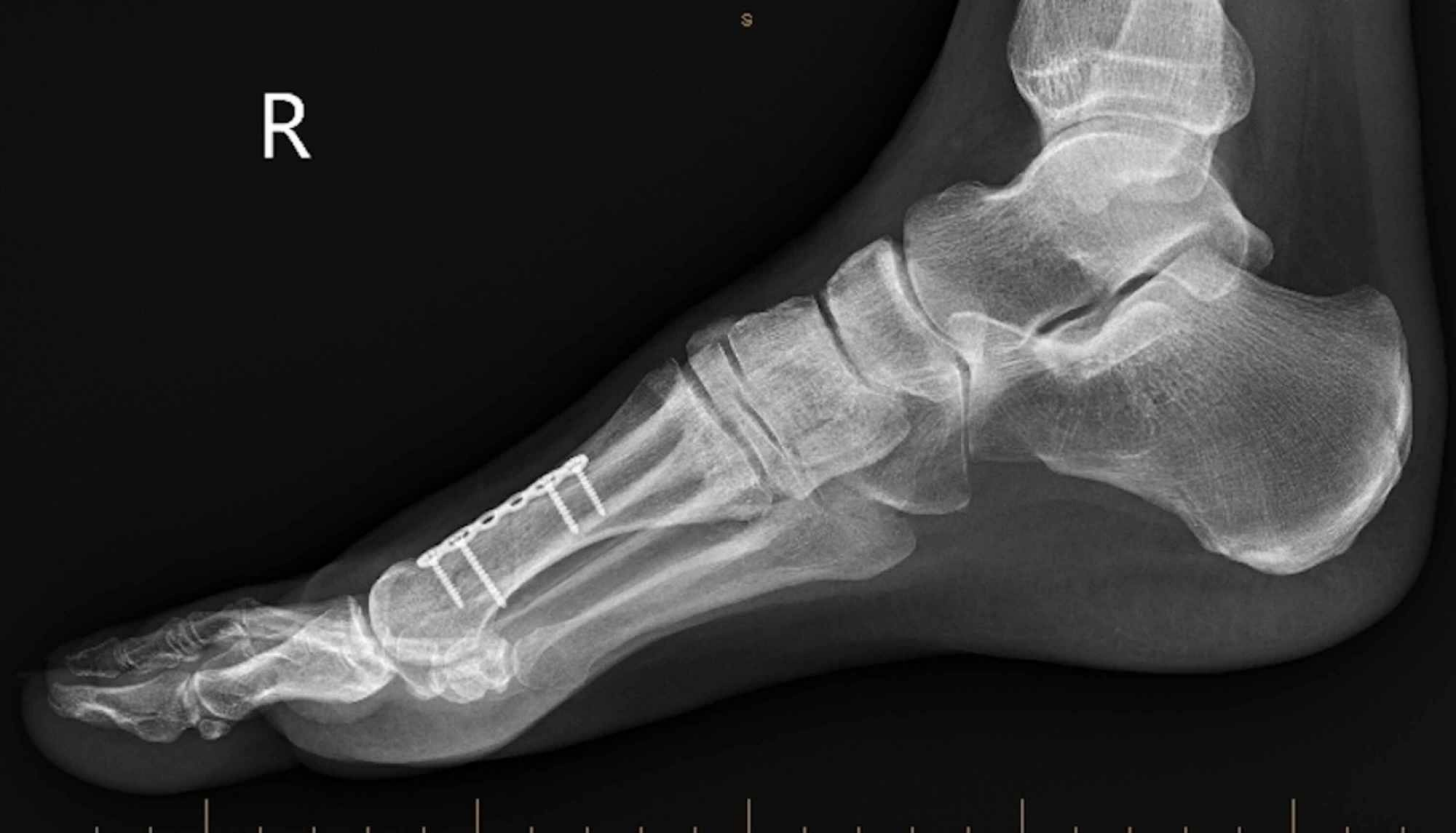
Post-operative lateral radiographs showing the return of the second metatarsal to its correct anatomical position

Post-operatively, the patient remained non-weight-bearing for three weeks. She was then able to resume partial weight-bearing for a further five weeks in a controlled ankle movement (CAM) walker boot with crutches. Rivaroxiban 10 mg one tablet daily for 10 days was instituted for venous thromboembolism (VTE) prophylaxis. Post-operative anterior-posterior and lateral radiographs show the reduction of the fracture and re-establishment of the metatarsal parabola (Figures 5, 6).

## Discussion

Metatarsal fractures are relatively common injuries, yet little is written about them in the academic literature. Fractures of the lesser metatarsals seem to occur more frequently than breaks of the first and are frequently fractured via direct trauma, with 9% sustained through sporting activities [7,11,12,13]. We suspect that their incidence is considerably higher than the reported statistics as most of these injuries would presumably go undocumented.

For such a common traumatic injury, the lack of information in the medical literature was a concern. We felt that it was important to provide some guidance for foot & ankle surgeons in the appropriate management of an isolated and misaligned lesser metatarsal fracture.

Whilst non-displaced or minimally-displaced second metatarsal fractures are usually amenable to non-operative treatment, surgical intervention may be required if there is a displacement of 3 mm or an angulation of 10 degrees or over. Sagittal plane displacement appears the least tolerated by patients and fractures of the shaft of the metatarsal seem to have a higher incidence of mal-union and displacement when compared to other types of metatarsal fractures [3,11].

The reality that the lesser metatarsals fracture with greater frequency when compared to the first is presumably due to their reduced diameter. The second metatarsal has a rigid ligamentous anchor between its head and the adjacent anatomic structures that tends to protect against displacement, yet in our case, the trauma was of sufficient force to cause significant misalignment [2,14].

Bone grafting in the surgical repair of this type of injury is often performed. Commonly, an autogenous bone graft harvested from the ipsilateral calcaneus or tibia may be employed into the fracture site. We anticipated that this may be required in our case, yet we were able to achieve fixation without grafting. Again, the site of graft harvesting is another area of controversy. Graft harvesting may be more appropriate in non- and mal-union fractures rather than in cases like ours.

Alternate methods of fixation have been reported. Sammarco and Carrasquillo reported two methods of fixation involving intramedullary reaming with Steinmann pin fixation, as well as an approach using double-threaded screw fixation through the MTPJ [15]. Russell used a compression bone plate with tibial bone graft to fixate a dorsal wedge osteotomy nonunion that had displaced [16].

In our case, the resultant misalignment of the metatarsal affected the sagittal plane only. We presume that multi-planar displacement would increase the complexity of the surgical repair and necessitate the need for some type of bone graft. We spent considerable time analysing the preoperative radiographs to decide on the declination angle of our osteotomy to ensure the correct reduction of the fracture in order to re-establish the metatarsal parabola. One of our concerns was that if the distal fragment was plantar flexed too much, then the patients ongoing third metatarsal plantar pain would be transferred with similar severity to the second metatarsal head, requiring further management. Another concern was that as the metatarsal head had remained in a dorsiflexed position for 12 months, the overlying extensor tendon had shortened, resulting in elevation of the second digit. Following ORIF, we performed an extensor lengthening procedure to return this digit to its correct alignment, and this digit was strapped into alignment for the first 3 weeks of the patient’s recovery.

Another area of controversy is the post-operative protocol. We elected to keep the patient non-weight-bearing in a fibre-glass back-slab for a period of three weeks, followed by partial weight-bearing in a CAM walker and crutches for a further 5. there is a discrepancy in the literature regarding metatarsal fracture post-operative protocols, yet we felt a conservative approach was warranted [5]. We elected to institute post-operative thromboprophylaxis given the patient’s sex and required convalescence in the back-slab.

Future research into the surgical intervention and post-operative protocols for metatarsal fractures is warranted and would benefit clinicians and surgeons.

## Conclusions

This case illustrates the surgical treatment of a second metatarsal shaft fracture that had dorsiflexed and shortened. The fracture was reduced via ORIF with a 6-hole 2.0 Osteomed® HPS locking plate. A long-arm extensor tendon lengthening was performed to reduce the elevated position of the second digit. This approach was able to re-establish the normal weight-bearing parabola of the metatarsal heads and to reduce the burden on the third MTPJ. Close postoperative monitoring was implemented. At the 12-month follow-up, the patient reported a complete resolution of symptoms with no recurrence of her third MTPJ pain. ORIF of dorsiflexed second metatarsal shaft fractures can provide reliable long-term outcomes.
